# Eye exercises of acupoints: their impact on refractive error and visual symptoms in Chinese urban children

**DOI:** 10.1186/1472-6882-13-306

**Published:** 2013-11-07

**Authors:** Zhong Lin, Balamurali Vasudevan, Vishal Jhanji, Tie Ying Gao, Ning Li Wang, Qi Wang, Ji Wang, Kenneth J Ciuffreda, Yuan Bo Liang

**Affiliations:** 1 The Affiliated Eye Hospital, School of Optometry and Ophthalmology, Wenzhou Medical University, 270 West College Road, Wenzhou 325027, Zhejiang, China; 2 Beijing Tongren Eye Center, Beijing Tongren Hospital, Capital Medical University; Beijing Ophthalmology & Visual Science Key Lab, Beijing, China; 3 College of Optometry, Mid Western University, Glendale, AZ, USA; 4 Department of Ophthalmology and Visual Sciences, The Chinese University of Hong Kong, Hong Kong, China; 5 Centre for Eye Research Australia, University of Melbourne, Parkville, Australia; 6 Handan Eye Hospital, Handan, Hebei, China; 7 Center for Studies in Constitution Research of Traditional Chinese Medicine, School of Basic Medicine, Beijing University of Chinese Medicine, Beijing, China; 8 Department of Biological and Vision Sciences, SUNY College of Optometry, New York, NY, USA

**Keywords:** Eye exercises, Acupoints, Myopia, Ocular fatigue, Near vision symptoms

## Abstract

**Background:**

Traditional Chinese eye exercises of acupoints involve acupoint self-massage. These have been advocated as a compulsory measure to reduce ocular fatigue, as well as to retard the development of myopia, among Chinese school children. This study evaluated the impact of these eye exercises among Chinese urban children.

**Methods:**

409 children (195 males, 47.7%), aged 11.1 ± 3.2 (range 6–17) years, from the Beijing Myopia Progression Study (BMPS) were recruited. All had completed the eye exercise questionnaire, the convergence insufficiency symptom survey (CISS), and a cycloplegic autorefraction. Among these, 395 (96.6%) performed the eye exercises of acupoints. Multiple logistic regressions for myopia and multiple linear regressions for the CISS score (after adjusting for age, gender, average parental refractive error, and time spent doing near work and outdoor activity) for the different items of the eye exercises questionnaire were performed.

**Results:**

Only the univariate odds ratio (95% confidence interval) for “seriousness of attitude” towards performing the eye exercises of acupoints (0.51, 0.33-0.78) showed a protective effect towards myopia. However, none of the odds ratios were significant after adjusting for the confounding factors. The univariate and multiple *β* coefficients for the CISS score were -2.47 (p = 0.002) and -1.65 (p = 0.039), -3.57 (p = 0.002) and -2.35 (p = 0.042), and -2.40 (p = 0.003) and -2.29 (p = 0.004), for attitude, speed of exercise, and acquaintance with acupoints, respectively, which were all significant.

**Conclusions:**

The traditional Chinese eye exercises of acupoints appeared to have a modest effect on relieving near vision symptoms among Chinese urban children aged 6 to 17 years. However, no remarkable effect on reducing myopia was observed.

## Background

Myopia in school children is a critical public health problem, especially in Asian countries [1-4]. After years of scientific research, the precise etiology of myopia remains elusive. Furthermore, there are no general and well-accepted guidelines followed by eye care practitioners for interventions that may decrease myopia development in children [5]. The Chinese eye exercises of acupoints, which commenced in the early 1960s, have been a compulsory measure during the school years introduced by the Chinese National Education Commission. These exercises are performed twice a day (morning and afternoon) by children in the school for the purpose of relieving ocular fatigue and reducing myopia. Despite the intervention, the prevalence of myopia and myopia-related visual impairments are on the rise in both urban and rural China [6,7].

**Figure 1:**
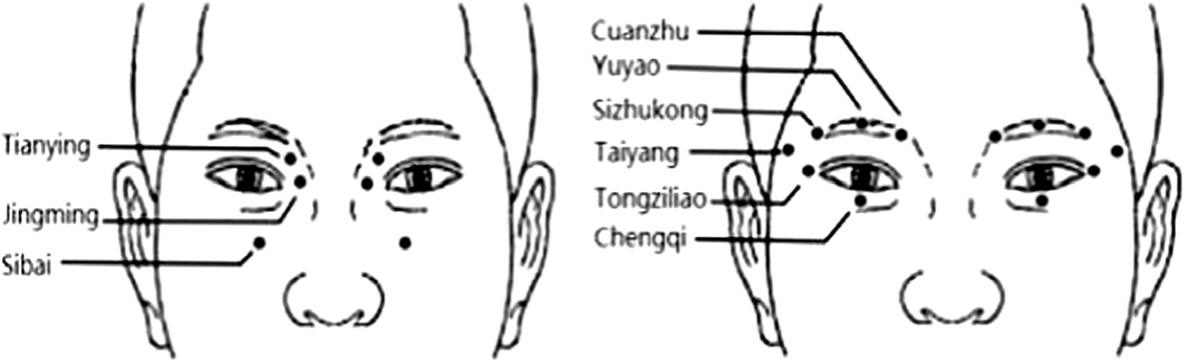
**Schematic diagram showing the positions of the acupoints used for eye exercises of acupoints.** Tianying: On the face, in the depression slightly below the medial end of the eyebrow; Jingming (BL1): On the face, in the depression slightly above the internal canthus; Sibai (ST2): On the face, directly below the pupil, in the depression of the infraorbital foramen; Taiyang (EX-HN5): At the temporal part of the head, between the lateral end of the eyebrow and the external canthus, in the depression one finger breadth behind them; Cuanzhu (BL2): On the face, in the depression of the medial end of the eyebrow, at the supraorbital notch; Yuyao (EX-HN4): On the forehead, at the midpoint of the eyebrow; Sizhukong (TE23): On the face, lateral to the external of eyebrow; Tongziliao (GB1): On the face, lateral to the external canthus, on the lateral border of the orbit; Chengqi (ST1): On the face, directly below the pupil, between the eyeball and the infraorbital ridge.

Acupuncture is a basic component of traditional medicine in East Asia, and it is also a popular 'alternative’ treatment modality in the West [8-10]. Fine needles are inserted into precisely defined points (acupoints) on the body to correct “disruptions in harmony” [10]. Randomized clinical trials reported that acupuncture therapy has some positive effect for dry eye [11], amblyopia [12,13], and myopia [14,15], although the effect of acupressure for slowing the progression of myopia is controversial, as reported by a recent Cochrane review [16]. The traditional Chinese eye exercises of acupoints requires approximately 5 minutes to perform. They involve bilateral acupoint self-massage that includes: (1) knead Tianying (Ashi) point, (2) press and squeeze Jingming (BL1), (3) press and knead Sibai (ST2), and (4) press Taiyang (EX-HN5) and scrape Cuanzhu (BL2), Yuyao (EX-HN4), Sizhukong (TE23), Tongziliao (GB1), Chengqi (ST1) (Figure 1). One epidemiological study (n = 612) reported that the prevalence of myopia was lower in primary students (grades 2–6) who performed these eye exercises of acupoints regularly as compared to students who performed these exercises infrequently (29.53% vs. 38.52%, *χ*^2^ = 6.04, p < 0.05) [17]. Recently, another study has demonstrated that these eye exercises of acupoints were protective (univariate odds ratio, 95% confidence interval: 0.605, 0.475-0.770) for juvenile myopia [18]. It has also been reported that having a “serious attitude” towards performing these eye exercises of acupoints improved visual acuity in primary school students [19]. However, the underlying mechanism of these eye exercise to reduce myopia remains elusive. One study did indicate that they improved local blood circulation as observed by color Doppler imaging [20].

Convergence insufficiency is associated with near visual symptoms, including eyestrain, headaches, blurred vision, diplopia, difficulty concentrating, and loss of comprehension after brief short periods of reading or performing near work activities [21-23]. The convergence insufficiency symptom survey (CISS) rating scale, a valid and reliable survey instrument for children [22], allows a two-factor analysis of the visual symptoms; firstly, whether or not the symptom is present, and secondly, how frequently the symptom occurs.

In the current study, the aim was to evaluate the impact of these eye exercises of acupoints on refractive error, as well as the near visual symptoms using the CISS questionnaire, among Chinese urban children (aged 6–17 years) recruited from the baseline population of the 3-year cohort Beijing Myopia Progression Study (BMPS) [24].

## Methods

### Subjects

A total of 409 children (195 males, 47.7%) from BMPS, who completed the eye exercises of acupoints questionnaire, the CISS questionnaire, and had a cycloplegic autorefraction, were included. Of these, 395 children (96.6%), 187 males and 208 females (47.3% and 52.7%), performed the eye exercises of acupoints in school. Details of the study design, sample size estimation, and baseline characteristics of BMPS have been reported elsewhere [24]. Briefly, children from elite primary or secondary schools in Beijing were recruited. The inclusion criteria were: (1) best-corrected visual acuity (BCVA) 0.1 (log minimum angle of resolution, LogMAR) or better; and (2) willing to cooperate and return for scheduled visits. The exclusion criteria were: (1) presence of amblyopia and/or strabismus; (2) history of intraocular surgery or ocular trauma; and (3) severe medical/ocular health problems.

The study followed the tenets of the Declaration of Helsinki and was approved by the Beijing Tongren Hospital Ethics Committee. All participants (children and their parents) provided signed and written informed consent.

### Questionnaire

Children were surveyed at the testing center with the help of trained staff regarding their habitual refraction, daily activities (indoor and outdoor), living environment, study pressure, study motivation, diet, eye exercises of acupoints, and the CISS questionnaire. For very young children who could not read or understand the questionnaire very well (e.g., primary students in grade 1), help was sought from their parent(s) to complete the questionnaire, which differs slightly from the CITT study [22].

#### *(1) Myopia questionnaire*

The myopia questionnaire used in the Sydney Myopia Study (questionnaire available at http://www.cvr.org.au/sms.htm) [25] was translated into Chinese with minor modifications. This questionnaire included the general topics of children’s habitual refraction, duration and type of daily activities, living environments, study pressure, study motivation, and diet [26]. The activities were grouped into near work and outdoor activities. The average hours spent on near work activity (<50 cm working distance) were summed from specific questions regarding drawing, homework, reading, and handheld computer use. Time spent on outdoor activities was based on questions regarding playing outdoors, family picnics and barbeques, bicycle riding, hiking, and outdoor sports.

#### *(2) Eye exercises of acupoints questionnaire*

The participants were asked to complete a self-designed eye exercises questionnaire (Additional file 1). The questionnaire consists of 11 items related to motivation, performance frequency, and attitude towards the eye exercises of acupoints.

#### *(3) Convergence insufficiency symptom survey (CISS)*

Studies from the Convergence Insufficiency Treatment Tail (CITT) group have demonstrated that the CISS is a valid instrument for quantifying near vision symptoms in 9 to 18 year-old children [22,27]. The CISS (Additional file 1) consists of 15 items with 5 response categories for each item [22]. It is scored as follows: never (0), infrequently (1), sometimes (2), fairly often (3), and always (4). The total score is obtained as a sum of scores for all 15 items (range from 0 to 60). Different from the CITT study, children of the current study completed the CISS with the help of trained staff, as well as their parent(s), rather than read it to the children exactly without assistance. Furthermore, 159 (159/409, 38.9%) of the children were less than 9-years-old, for which the CISS questionnaire has not been validated yet.

### Refractive error

All children received a cycloplegic autorefraction (*Accuref-K9001, Shin Nippon, Japan*), whereas the parents received a non-cycloplegic autorefraction due to their age and related markedly reduced accommodation. Cycloplegic autorefraction was performed after instilling 3 drops of cyclopentolate 1% (Cyclogyl, Alcon). Three readings were obtained for each eye in all participants, and the data were averaged for each subject, and then averaged across each group.

### Data analysis

Due to high correlation of the cycloplegic refractive error (spherical equivalent, SE) between the right and left eyes (Pearson correlation coefficient 0.95, p < 0.001), only the SE of right eye of each student was used in the analysis. Myopia was defined as SE ≤ -0.50 D [24]. Parental refractive error was defined as the average of the non-cycloplegic SE for each eye for the combined father and mother [26].

Both univariate and multiple (after adjusting for children’s age, gender, average parental refractive error, near work, and outdoor activity hours) logistic regressions for myopia involving the different items of the eye exercises of acupoints questionnaire were performed. Univariate and multiple (adjusting for the same potential confounding factors) linear regressions for the CISS score involving the different items of the eye exercises of acupoints were also performed.

Children were further divided into four groups after pair-wise combination of the attitude towards the eye exercises of acupoints (serious or not) and acquaintance with the acupoints (acquaintance or not). These groups were: Group 1: neither seriously performing the eye exercises nor acquainted with the acupoints; Group 2: seriously performed the exercises but not acquainted with the acupoints; Group 3: not serious about the eye exercises but acquainted with the acupoints; Group 4: seriously performed the eye exercises and acquainted with the acupoints. The SE and CISS score among these groups were compared using one-way analysis of variance (ANOVA). Multiple logistic regressions for myopia were also calculated with group 1 as the reference group.

## Results

The mean age of the children was 11.1 ± 3.2 (range 6–17) years. The overall mean SE and CISS scores were -1.70 ± 2.38 D and 13.0 ± 7.9 for children who performed the eye exercises of acupoints (n = 395), and -1.60 ± 1.71 D and 14.3 ± 9.8 for children who did not (n = 14). No significant difference was found either in the SE or the CISS scores between these two groups of children (t = 0.15, p = 0.88; t = 0.60, p = 0.55, respectively). Table 1 presents the distribution of the children’s responses for each item of the eye exercises of acupoints questionnaire. There were 260 (65.8%) and 127 (32.2%) children who performed the eye exercises of acupoints for relief of ocular fatigue and were required to do so by either their teachers or parents, respectively. Seven (1.8%) children reported performing the eye exercises of acupoints to protect their visual acuity, and 1 (0.2%) student did not specify any reason for doing so.

**Table 1:** Distribution of children’s responses for each item of the eye exercises of acupoints questionnaire

	**Number (%)**
Perform eye exercises of acupoints (in school)	
No	14 (3.4)
Yes	395 (96.6)
Purpose (in school)	
Relieving ocular fatigue	260 (65.8)
Required by teachers or parents	127 (32.2)
Other reason	8 (2.0)
Times per day (in school)	
< 2	56 (14.2)
≥ 2	339 (85.8)
Serious or not	
No/moderate	216 (54.7)
Yes	179 (45.3)
Serious times per week	
None	34 (8.6)
< 3	78 (19.8)
≥ 5	283 (71.6)
Eye exercises of acupoints were taught by	
Atlas/classmate	60 (15.2)
Teacher/doctor/healthy counselor	335 (84.8)
Speed	
Faster/slower than the broadcast & at will	60 (15.2)
Following the broadcast	335 (84.8)
Acquaintance of eye exercises acupoints	
No/moderate	237 (60.0)
Yes	158 (40.0)
Perform additional eye exercises of acupoints (outside school)	
No	271 (68.6)
Yes	124 (31.4)
Purpose (outside school)	
Relieving ocular fatigue	98 (79.0)
Required by teachers or parents	19 (15.3)
Other reason	7 (5.7)
Times per day (outside school)	
< 2	38 (30.6)
≥ 2	86 (69.4)

Table 2 presents the student’s SE, univariate and multiple odds ratio (OR), and 95% confidence interval (CI) for myopia, for each item of the eye exercises of acupoints questionnaire. The SE was significantly less myopic in the children who performed the exercises seriously as compared to those who did not (-1.36 ± 2.31 D vs. -1.98 ± 2.40 D; t = -2.62, p = 0.009). Performing the eye exercises of acupoints seriously initially also revealed a significant protective effect for myopia (univariate OR, 95% CI: 0.51, 0.33-0.78). However, the OR was not significant after adjusting for children’s age, gender, parental refractive error, and time spend on near work and outdoor activity (OR, 95% CI: 0.71, 0.41-1.24). Children who performed additional eye exercises outside of the school hours (n = 124) had a significantly more myopic SE as compared to those who did not (n = 271) (-2.15 ± 2.44 D vs. -1.49 ± 2.32 D; t = 2.56, p = 0.01). Furthermore, both the univariate (OR, 95% CI: 1.58, 0.98-2.57) and multiple OR (OR, 95% CI: 1.82, 0.99-3.34) for myopia approached significance in this group of children.

**Table 2:** Children’s spherical equivalent and odds ratio for myopia by each item of the eye exercises of acupoints questionnaire

	**SE (mean ± SD)**	**Univariate OR (95% CI)**	Multiple OR (95% CI)^*^	Multiple OR (95% CI)^*^
Performed eye exercises of acupoints (in school)				
No	-1.60 ± 1.71			
Yes	-1.70 ± 2.38	0.62 (0.17, 2.25)	1.91 (0.37, 9.76)	1.46 (1.32, 1.63)
Times per day (in school)				
< 2	-1.92 ± 2.28			
≥ 2	-1.66 ± 2.39	0.58 (0.29, 1.13)	0.61 (0.26, 1.45)	1.51 (1.35, 1.69)
Serious or not				
No/moderate	-1.98 ± 2.40			
Yes	-1.36 ± 2.31^†^	0.51 (0.33, 0.78)	0.71 (0.41, 1.24)	1.50 (1.34, 1.68)
Serious times per week				
None	-1.85 ± 1.73			
< 3	-1.45 ± 2.53	0.58 (0.22, 1.52)	1.08 (0.33, 3.50)	
≥ 5	-1.75 ± 2.40	0.56 (0.23, 1.32)	0.94 (0.32, 2.73)	1.51 (1.35, 1.70)
Eye exercises of acupoints were taught by				
Atlas/classmate	-1.77 ± 2.66			
Teacher/doctor/healthy counselor	-1.69 ± 2.32	0.97 (0.53, 1.76)	1.16 (0.55, 2.46)	1.51 (1.35, 1.70)
Speed				
Faster/slower than the broadcast & At will	-1.70 ± 1.80			
Following the broadcast	-1.70 ± 2.47	0.61 (0.32, 1.17)	0.80 (0.35, 1.85)	1.51 (1.35, 1.69)
Acquaintance of eye exercises acupoints				
No/Moderate	-1.85 ± 2.40			
Yes	-1.48 ± 2.32	0.92 (0.60, 1.43)	1.11 (0.64, 1.95)	1.51 (1.35, 1.70)
Perform additional eye exercises of acupoints (outside school)				
No	-1.49 ± 2.32			
Yes	-2.15 ± 2.44^†^	1.58 (0.98, 2.57)	1.82 (0.99, 3.34)	1.51 (1.35, 1.70)

SE: spherical equivalent; SD: standard deviation; OR: odds ratio; CI: confidence interval; the first group was the reference group.

Multiple OR^*^: adjusted for children’s age, gender, average parental refractive error, times spend on near work and outdoor, and item of eye exercises questionnaire; column 3 presented the OR (95% CI) of item of eye exercises questionnaire; column 4 presented the OR (95% CI) of item of children’s age.

^†^: significantly different as compared to the first group.

Table 3 presents the CISS score, as well as univariate and multiple *β* coefficients of the CISS score, for each item of the eye exercises of acupoints questionnaire. The CISS score was significantly less in those children who performed the exercises seriously as compared to those who did not (11.6 ± 8.5 vs. 14.1 ± 7.3; t = -3.06, p = 0.002). A more serious attitude towards the exercises was also significantly associated with a lower CISS score (univariate *β = -*2.47, p = 0.002), *even* after adjusting for student’s age, gender, refractive error, average parental refractive error, and time spend on near work and outdoor activity (multiple *β = -*1.65, p = 0.039). This score was significantly less in children who followed the school broadcast when doing the exercises as compared to those who did not (12.5 ± 7.8 vs. 16.0 ± 8.2; t = -3.19, p = 0.002). Both the univariate and multiple *β* coefficients were significant (*β = -*3.57, p = 0.002; *β = -*2.35, p = 0.042). The CISS score was significantly less in children who were acquainted with the acupoints as compared to those who were not (11.5 ± 8.4 vs. 13.9 ± 7.5; t = 2.97, p = 0.003). Both the univariate and multiple *β* coefficients were significant (*β = -*2.40, p = 0.003; *β = -*2.29, p = 0.004). Similar results were yielded with student’s refractive error further adjusted.

**Table 3:** **Children’s convergence insufficiency symptom survey scores and *β *** **coefficients for each item of eye exercises of acupoints questionnaire**

	**CISS score (mean ± SD)**	**Univariate *β *** **coefficient (p value)**	**Multiple *β *** coefficient (p value)^*^	**Multiple *β *** coefficient (p value)^**^	**Multiple *β *** coefficient (p value)^**^
Performed eye exercises of acupoints (in school)					
No	14.3 ± 9.8				
Yes	13.0 ± 7.9	-1.34 (0.55)	4.39 (0.10)	4.08 (0.13)	0.75 (<0.0001)
Times per day (in school)					
< 2	13.5 ± 7.4				
≥ 2	12.9 ± 8.0	-0.66 (0.57)	-1.35 (0.23)	-1.30 (0.25)	0.78 (<0.0001)
Serious or not					
No/moderate	14.1 ± 7.3				
Yes	11.6 ± 8.5^†^	-2.47 (0.002)	-1.65 (0.039)	-1.62 (0.045)	0.75 (<0.0001)
Serious times per week					
None	12.8 ± 8.1				
< 3	15.0 ± 7.6				
≥ 5	12.4 ± 7.9	-0.89 (0.16)	-0.55 (0.38)	-0.59 (0.35)	0.77 (<0.0001)
Eye exercises of acupoints were taught by					
Atlas/classmate	13.2 ± 8.3				
Teacher/doctor/healthy counselor	12.9 ± 7.9	-0.26 (0.82)	-0.14 (0.90)	-0.19 (0.86)	0.78 (<0.0001)
Speed					
Faster/slower than the broadcast/at will	16.0 ± 8.2				
Following the broadcast	12.5 ± 7.8^†^	-3.57 (0.002)	-2.35 (0.042)	-2.42 (0.037)	0.77 (<0.0001)
Acquaintance of eye exercises acupoints					
No/moderate	13.9 ± 7.5				
Yes	11.5 ± 8.4^†^	-2.40 (0.003)	-2.29 (0.004)	-2.26 (0.005)	0.76 (<0.0001)
Perform additional eye exercises of acupoints (outside school)					
No	13.1 ± 7.6				
Yes	12.8 ± 8.6	-0.30 (0.73)	-0.80 (0.34)	-0.99 (0.24)	0.77 (<0.0001)

CISS: convergence insufficiency symptom survey; SD: standard deviation.

Multiple *β* coefficient^*^: adjusted for children’s age, gender, average parental refractive error, times spend on near work and outdoor, and item of eye exercises questionnaire; column 3 presented the OR (95% CI) of item of eye exercises questionnaire.

Multiple *β* coefficient^**^: adjusted for children’s age, gender, refractive error, average parental refractive error, times spend on near work and outdoor, and item of eye exercises questionnaire; column 4 presented the OR (95% CI) of item of eye exercises questionnaire; column 5 presented the OR (95% CI) of item of the children’s age.

^†^: significantly different as compared to the first group.

**Figure 2:**
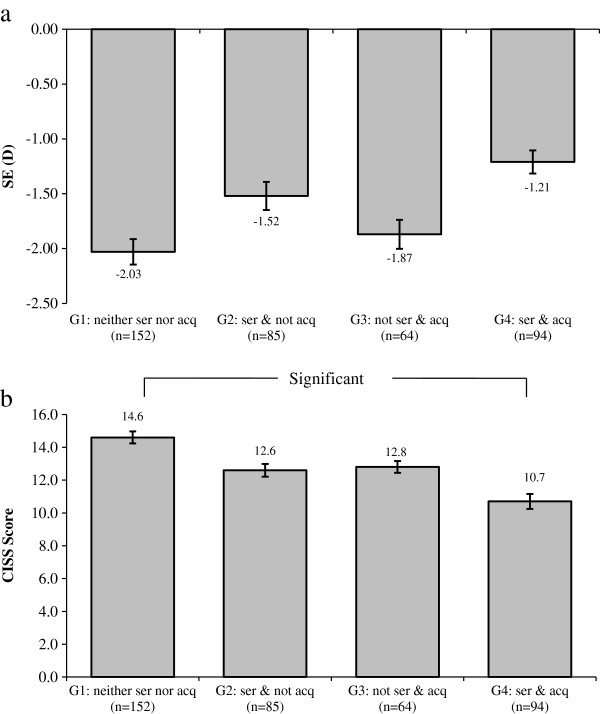
**Spherical equivalent (SE, diopters) and convergence insufficiency symptom survey (CISS) in different groups. a** SE of the different groups by attitude towards performing the eye exercises of acupoints (seriously or not) and acupoint acquaintance level (acquainted or not). The SE demonstrated a significant trend among different groups (p=0.05). Ser: performed eye exercises of acupoints seriously; acq: acquainted with acupoints. Plot is the mean ± standard error. **b** CISS score of the different groups by attitude towards performing the eye exercises of acupoints (seriously or not) and acupoint acquaintance level (acquainted or not). The score was significantly different among the groups (p=0.002), namely between the first and last group (bonferroni corretion). ser: performed eye exercises of acupoints seriously; acq: acquainted with eye exercises acupoints. Plot is the mean ± standard error.

**Figure 3:**
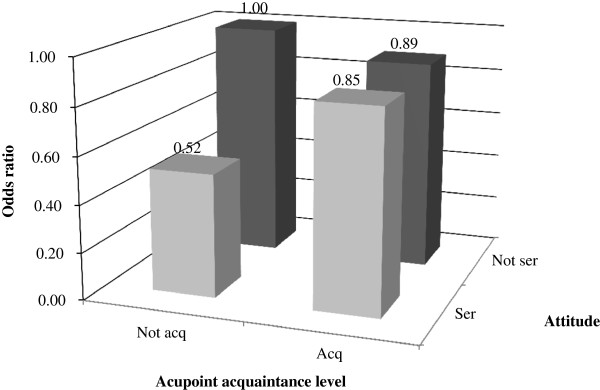
**Multiple-adjusted odds ratios (adjusted for age, gender, average parental refractive error, time spent on near work and outdoor activity) for myopia by attitude towards performing the eye exercises of acupoints (seriously or not) and acupoint acquaintance level (acquainted or not).** The group with a more serious attitude for performing the eye exercises of acupoints and acupoint acquaintance of eye exercises was the reference group. ser: performed eye exercises of acupoints seriously; acq: acquainted with eye exercises acupoints.

Children were further divided into four groups after pairwise combination of the attitude towards the eye exercises of acupoints (serious or not) and acquaintance with acupoints (acquainted or not). The SE demonstrated a significant trend among the 4 groups (F = 2.59, p = 0.05). The SE tended to be progressively less myopic from Group 1 (-2.03 ± 2.31) to Group 4 (-1.21 ± 2.08) (Figure 2a). The CISS score was significantly different among the 4 groups (F = 5.00, p = 0.002), and the post-hoc test (bonferroni correction) showed a significant difference between Group 1 and Group 4 (Figure 2b). The combined effects of performing the eye exercises of acupoints seriously and being acquainted with the acupoints on the odds of myopia are presented in Figure 3. Group 1 was used as the reference group (OR, 1.00). Although lower odds for myopia were found in the other 3 groups, none of them was statistically significant (Figure 3).

## Discussion

To the best of our knowledge, this is the first study that provides detailed information on the effects of traditional Chinese eye exercises of acupoints in school children. There was one key finding in the current study. The seriousness of attitude towards performing these eye exercises and acquaintance with the acupoints were associated with a lower CISS score, i.e., less ocular-based near vision symptoms.

Less myopic refractive error was observed in children who performed the eye exercises of acupoints seriously as compared to those who did not. However, this protective effect was lost after two significant confounders, namely age and parental refractive error, were adjusted. Furthermore, refractive error of the children did not tend to be relatively more hyperopic as time for performing the eye exercises of acupoints per week increased. This finding may undermine the protective effect of the eye exercises of acupoints on the reduction of myopic refractive error.

The refractive error was significantly more myopic in children who performed the *additional* eye exercises of acupoints after school hours. There could be two possible reasons underlying this unexpected finding. First, children with a more myopic refractive error may have had greater determination, or were under greater psychological pressure, to do so from their teachers and/or parents to draw less attention to the problem of myopic progression, and furthermore to prevent yet further increases. Second, performing the eye exercises of acupoints after school involved less actual time than when performed in school (percentage of those performing the eye exercises of acupoints twice per day or more: 69.4% vs. 85.8%). However, quality of the eye exercises of acupoints performed outside of school hours could not be guaranteed without supervision.

In the present study, the prevalence of myopia was not associated either with one’s attitude towards performing the eye exercises of acupoints or acquaintance with the related acupoints. However, these results should be considered along with certain limitations of the study design. First, as mentioned earlier, children with a greater myopic refractive error may be more determined to perform the eye exercises of acupoints, and they may also have a better understanding of the eye exercises’ acupoints. Second, urban Chinese children are exposed to high risk factors for causation of myopia, such as increased near work activity hours, less outdoor activity hours, a crowded living environment, and a higher probability of having myopic parents [28,29]. Thus, the overall effect of these eye exercises on reduction of refractive error by only 5–10 minutes of exercise time each day in this highly susceptible population appeared to be negligible. Longer and more intensive eye exercises of acupoints may thus be necessary to be effective. Hence, well-designed randomized controlled trial (RCTs) would be necessary to investigate the effect of different durations of the eye exercises of acupoints on reducing myopia.

In the current study, children who performed the eye exercises of acupoints seriously, who followed the school broadcast when performing the exercises, and who were acquainted with the exercises acupoints, tended to have a lower CISS score even after adjusting for the confounders. Considering the linkage and correlation between accommodation, vergence, and refractive error [30,31], a further multiple regression model with children’s refractive error adjusted was employed, with it yielding similar results. However, the reduction in symptoms was relatively small (CISS score reduced 2.5-3.5 points). Borsting et al. reported that the CISS score was 30.8 in children with convergence insufficiency; children with a CISS score of 16 or higher are considered to be symptomatic [22]. The CISS score of the current study was approximately 14, consequently less than in children with convergence insufficiency. Hence, there may be a floor effect for the CISS score to reduce yet further. Our current findings suggested that the eye exercises of acupoints modestly reduced these ocular-based near symptoms in Chinese urban children with high normal scores. It would be interesting to determine if the eye exercises of acupoints would have more effect on relief of near symptoms in children with convergence insufficiency or a higher CISS score. Furthermore, the placebo effect of the eye exercises may not be excluded, since there was no control group in the current study.

The eye exercises of acupoints mildly reduced the ocular-based near symptoms. However, little is currently known about the mechanisms underlying reduction of these symptoms. It has been proposed that acupuncture at these vision-related acupoints increased blood flow to the cerebral and ocular vasculatures (including the choroid) [32-34], thus leading to metabolic changes in the central and peripheral nervous system [34-36]. Although the effect of such massage in these vision-related acupoints may be less than acupuncture itself, it is likely that the effects of the eye exercises of acupoints on visual symptoms is due to multiple mechanisms. In addition, simple cessation of near work to perform the eye exercises of acupoints provides a brief rest period that itself may reduce the visual fatigue effects [37].

There are potential limitations of the present study. First, the children were enrolled consecutively at the baseline of the Beijing Myopia Progression Study. Hence, results of the current study may not be generalisable to the entire population of Beijing children across the educational spectrum. Longitudinal data would provide more convincing evidence. Second, the results of this study would be stronger, with either a control or comparative group. Finally, further studies, especially well-designed RCTs, with a larger sample size and different eye exercise schedules, are warranted to understand better the dose-effect of these eye exercises of acupoints on myopia and the related visual-based near symptoms.

## Conclusions

This cross-sectional study demonstrated that the eye exercises of acupoints had a modest effect on relieving ocular-based near vision symptoms in Chinese urban children aged 6 to 17 years. However, no remarkable effect on reducing myopia was observed in this study.

## Competing interests

The authors declare that they have no competing interests.

## Authors’ contributions

YBL designed the study protocol and conducted the study as a supervisor. ZL, BV and KJC participated in the study design, conducted statistical analysis, and drafted the manuscript. KJC, BV, VJ, NLW, TYG and YBL participated in the study design, and revised the manuscript. QW and JW conducted the eye exercises of acupoints and revised the manuscript. All authors read and approved the final manuscript.

## Pre-publication history

The pre-publication history for this paper can be accessed here:

http://www.biomedcentral.com/1472-6882/13/306/prepub

## Supplementary Material

Additional file 1 **Appendix I.** Eye exercises of acupoints questionnaire. **Appendix II.** Convergence insufficiency symptom survey. Click here for file
