# Supplementary material for: Eye exercises of acupoints: their impact on refractive error and visual symptoms in Chinese urban children
Source: BMC Complement Altern Med. 2013 Nov 7;13:306. doi: 10.1186/1472-6882-13-306 (PMC3828420; doi:10.1186/1472-6882-13-306)
Supplement: Additional file 1 — Appendix I. Eye exercises of acupoints questionnaire. Appendix II. Convergence insufficiency symptom survey. [file 1472-6882-13-306-S1.docx]

**Additional file 1: Appendix I: eye exercises of acupoints questionnaire**

1. Do you perform eye exercises of acupoints in school?

□Yes □No

2. What is your reason for performing eye exercises of acupoints?

□Relieving ocular fatigue □Required by teachers or parents

□Other reason, please specify: _______________________

3. How often do you perform eye exercises of acupoints in school?

□More than twice a day □Twice a day □Once a day □Less than once a day

4. Do you think you perform eye exercises of acupoints seriously?

□Yes □Moderate □No

5. How many times do you perform eye exercises of acupoints seriously per week?

□None □Less than 3 times □More than 5 times

□Every time

6. Who taught you how to perform eye exercises of acupoints?

□School teacher(s) □Learn from health atlas

□Doctor(s) or school doctor(s) □Learn from classmate(s)

7. How fast do you perform eye exercises of acupoints?

□Faster than the broadcast □Follow the broadcast

□Slower than the broadcast □At will

8. Are you acquainted with the acupoints of eye exercises of acupoints?

□Yes □Moderate □No

9. Do you perform eye exercises of acupoints outside the school hours?

□Yes □No (completed the questionnaire here)

10. How often do you perform eye exercises of acupoints outside the school hours?

□More than twice a day □Twice a day □Once a day □Less than once a day

11. What is your reason of performing eye exercises of acupoints outside the school hours?

□Relieving ocular fatigue □Required by teachers or parents

□Other reasons, please specify: _______________________

**Additional file 1: Appendix II: convergence insufficiency symptom survey**

|  |  | **Never** | **Infrequently** | **Sometimes** | **Fairly often** | **Always** |
| --- | --- | --- | --- | --- | --- | --- |
| 1. | Do your eyes feel tired when reading or doing close work? |  |  |  |  |  |
| 2. | Do your eyes feel uncomfortable when reading or doing close work? |  |  |  |  |  |
| 3. | Do you have headaches when reading or doing close work? |  |  |  |  |  |
| 4. | Do you feel sleepy when reading or doing close work? |  |  |  |  |  |
| 5. | Do you lose concentration when reading or doing close work? |  |  |  |  |  |
| 6. | Do you have trouble remembering what you have read? |  |  |  |  |  |
| 7. | Do you have double vision when reading or doing close work? |  |  |  |  |  |
| 8. | Do you see the words move, jump, swim or appear to float on the page when reading or doing close work? |  |  |  |  |  |
| 9. | Do you feel like you read slowly? |  |  |  |  |  |
| 10. | Do your eyes ever hurt when reading or doing close work? |  |  |  |  |  |
| 11. | Do your eyes ever feel sore when reading or doing close work? |  |  |  |  |  |
| 12. | Do you feel a "pulling" feeling around your eyes when reading or doing close work? |  |  |  |  |  |
| 13. | Do you notice the words blurring or coming in and out of focus when reading or doing close work? |  |  |  |  |  |
| 14. | Do you lose your place while reading or doing close work? |  |  |  |  |  |
| 15. | Do you have to re-read the same line of words when reading? |  |  |  |  |  |
|  |  | x 0 | x 1 | x 2 | x 3 | x 4 |

**TOTAL SCORE**______________
